# A *CACNA1C* variant associated with cardiac arrhythmias provides mechanistic insights in the calmodulation of L-type Ca^2+^ channels

**DOI:** 10.1016/j.jbc.2022.102632

**Published:** 2022-10-21

**Authors:** Juan Zhao, Emilie Segura, Mireille Marsolais, Lucie Parent

**Affiliations:** 1 Centre de recherche de l’Institut de Cardiologie de Montréal, Université de Montréal, Montréal, Québec, Canada; 2 Département de Pharmacologie et Physiologie, Faculté de Médecine, Montréal, Québec, Canada

**Keywords:** protein–protein interaction, electrophysiology, coimmunoprecipitation, phosphorylation, window current, calmodulin inhibitors, CaM, calmodulin, CDI, Ca^2+^-dependent inactivation, cDNA, complementary DNA, CK2, casein kinase II, CMV, cytomegalovirus, HEK, human embryonic kidney cell line, LQTS, long-QT syndrome, TBB, 4,5,6,7-tetrabromobenzotriazole, TS, Timothy syndrome

## Abstract

We recently reported the identification of a *de novo* single nucleotide variant in exon 9 of *CACNA1C* associated with prolonged repolarization interval. Recombinant expression of the glycine to arginine variant at position 419 produced a gain in the function of the L-type Ca_V_1.2 channel with increased peak current density and activation gating but without significant decrease in the inactivation kinetics. We herein reveal that these properties are replicated by overexpressing calmodulin (CaM) with Ca_V_1.2 WT and are reversed by exposure to the CaM antagonist W-13. Phosphomimetic (T79D or S81D), but not phosphoresistant (T79A or S81A), CaM surrogates reproduced the impact of CaM WT on the function of Ca_V_1.2 WT. The increased channel activity of Ca_V_1.2 WT following overexpression of CaM was found to arise in part from enhanced cell surface expression. In contrast, the properties of the variant remained unaffected by any of these treatments. Ca_V_1.2 substituted with the α-helix breaking proline residue were more reluctant to open than Ca_V_1.2 WT but were upregulated by phosphomimetic CaM surrogates. Our results indicate that (1) CaM and its phosphomimetic analogs promote a gain in the function of Ca_V_1.2 and (2) the structural properties of the first intracellular linker of Ca_V_1.2 contribute to its CaM-induced modulation. We conclude that the *CACNA1C* clinical variant mimics the increased activity associated with the upregulation of Ca_V_1.2 by Ca^2+^–CaM, thus maintaining a majority of channels in a constitutively active mode that could ultimately promote ventricular arrhythmias.

Cardiac contraction during the systole is handled by the influx of Ca^2+^ into cardiomyocytes in response to depolarization during phase 2 of the cardiac action potential (1). Voltage-gated L-type calcium channel Ca_V_1.2 are expressed in the T-tubules such that localized Ca^2+^ entry triggers a sustained and more global Ca^2+^ release by the sarcoplasmic reticulum in the dyadic cleft (2). Cardiac L-type Ca_V_1.2 channels are heteromultimeric protein complexes formed by the pore-forming Ca_V_α1C subunit bound to the extracellular Ca_V_α2δ1 auxiliary subunits (3, 4) and to the cytoplasmic Ca_V_β (5) that binds with nanomolar affinity to the first intracellular linker (6). The Ca_V_α1 subunit is formed by a single polypeptide chain of 24 transmembrane helices grouped into four structural homologous domains (domains I, II, II, and IV) (Fig. 1). Although not a specific auxiliary subunit, calmodulin (CaM) contributes to Ca^2+^-dependent facilitation and Ca^2+^-dependent inactivation (CDI) of Ca_V_1.2 (7, 8, 9) through binding to the isoleucine–glutamine motif in the C-terminal tail of Ca_V_α1C (10, 11, 12, 13).

**Figure 1:**
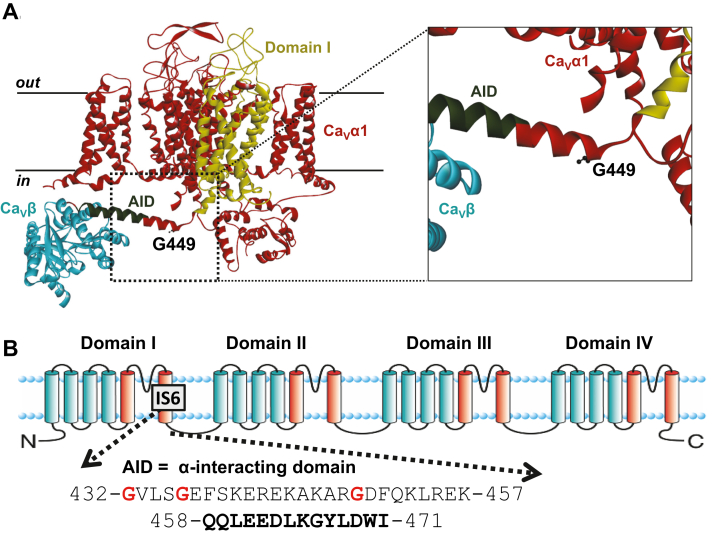
**The Ca**_**V**_**1.2 variant is located in the intracellular linker before the binding site for the Ca**_**V**_**β subunit.** The LQTS-related Ca_V_α1C mutation G449 is located before the α-interacting domain (AID). *A*, the cryo-EM 3D structure of the rabbit Ca_V_1.1 oligomeric complex at 3.6 Å for Ca_V_α1S and at 3.9 Å for Ca_V_β (Protein Data Bank code: 5GJV). Ca_V_α1C and Ca_V_α1S share 81% homology in their primary protein sequence. L-type calcium channels share similar structure, being composed of the pore-forming subunit Ca_V_α1 in *red* and Ca_V_β in *blue* and an intracellular subunit bound to Ca_V_α1 through the intracellular helix linking domains I and II of Ca_V_α1 (shown in *dark green*). The first transmembrane domain of Ca_V_α1 (DI) is shown in *yellow*. The human LQTS-related Ca_V_α1C G419R variant is similar to the rabbit Ca_V_α1C G449R and is equivalent to Gly358 in Ca_V_α1S. Image was produced by Discovery Studio 2020 (BIOVIA Pipeline Pilot 2020). *B*, cartoon of the corresponding secondary structure for the Ca_V_α1C pore-forming subunit of the L-type Ca_V_1.2 channel showing the four homologous domains (domains I to IV) with the N and C termini located into the cytoplasm. The Ca_V_β subunit–binding site on the Ca_V_α1C subunit is referred to the “α-interacting domain” or AID. The AID is located within 20 residues of the sixth transmembrane segment in domain I (IS6). The primary sequence for the AID motif is shown below the primary sequence for the short region extending from the end of S6 to the beginning of the AID. The relative position of three glycine variants reported in the Timothy syndrome (G402S, G406R, and G419R) is fully conserved across species and presented in *red* with the numbering in the rabbit clone used for this study. LQTS, long-QT syndrome.

First clinically described in 1957 (14), the long-QT syndrome (LQTS) is a major cause of sudden death in healthy infants and young adults (15, 16, 17). Congenital LQTS in the absence of structural defects (18) is often the result of inherited or *de novo* genetic mutations in the DNA of a variety of ion channels (19). Gain-of-function mutations within the *CACNA1C* gene, coding for Ca_V_α1C, are associated with the LQTS type 8 also referred to as Timothy syndrome (TS) (20, 21, 22). Many TS variants were identified in a short region adjoining the sixth transmembrane segment of the Ca_V_α1C protein (Fig. 1). The canonical TS1 variant Gly406Arg results from a *de novo CACNA1C* mutation in exon 8A (20). An atypical form of TS type 2 is associated with the Gly402Ser and Gly402Arg variants in the alternatively spliced exon 8 (21). More recent *de novo* mutations have highlighted the importance of this region such as Glu407Gly/Ala (23, 24) and Arg518Cys/His (25). These missense variants are causing a gain of function in the Ca_V_1.2 channel as a result of slower inactivation kinetics that promote larger Ca^2+^ influx for the same depolarizing pulse (26). Nonetheless, functional outcomes of other TS mutations included marked loss of current density, a gain-of-function shift in activation, and increased window current (27). We have recently identified in the first intracellular region of Ca_V_α1C a missense variant, Gly419Arg, from a patient with prolonged QT interval (≈500 ms), syndactyly, left ventricular noncompaction, and slight delay in neurodevelopment (28). Unlike other TS variants located close to the high-affinity binding domain of Ca_V_β, Ca_V_1.2 Gly419Arg exhibited a gain-of-function shift in the activation gating and no decrease in the channel current decay (28).

Herein, we explored the regulation of the long QTS variant Gly419Arg (G449R in the rabbit clone numbering). Glycine residues, inserted between the sixth transmembrane segment and the high-affinity binding site for Ca_V_β, have been shown to confer higher flexibility to this region (29, 30, 31), leading to reduced basal L-type Ca_V_ channel activity in cardiomyocytes (30). The reverse proposition, removing or substituting glycine residues in this locus, decreased the linker flexibility (31). Herein, we present evidence that the novel variant, whereby a conserved glycine is substituted by a larger arginine residue, promotes stronger activity (peak current density and activation gating) at physiological voltages akin to a constitutively hyperactive channel. This hyperactive mode was reconstituted in the WT channel by coexpression with CaM WT or pseudophosphorylated surrogates CaM T79D or CaM S81D and was abolished by the CaM antagonist W-13. In contrast, the functional parameters of the clinical glycine to arginine variant remained remarkably insensitive to these treatments. Substitution with the α-helix breaker proline residue yielded opposite results with channels more reluctant to open at physiological voltages but more likely to respond to the modulation by CaM. Altogether, the functional characterization of the glycine to arginine variant provides mechanistic insight on the regulation of Ca_V_1.2 by CaM (sometimes referred to as calmodulation) and specifically the role played by the I–II linker as relaying the signal to the channel activation gate.

## Results

### Glycine substitution stimulates activation gating and peak current density of Ca_V_1.2

**Figure 2:**
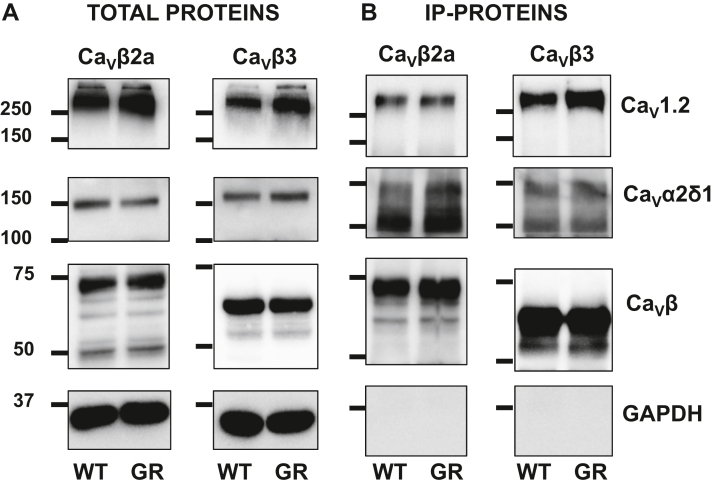
**G449R interacts with Ca**_**V**_**β proteins.** HEKT cells were transiently transfected with Ca_V_1.2 WT (WT) or Ca_V_1.2 G449R (GR) with cMyc-tagged Ca_V_β3 or cMyc-tagged Ca_V_β2a. Ca_V_α2δ1 was present throughout. Cell lysates were immunoprecipitated overnight with anti-cMyc magnetic beads (Pierce Anti-c-Myc Magnetic Beads; catalog no.: 88842, Thermo Fisher Scientific) to capture the Ca_V_β, eluted in a 2× Laemmli buffer and fractionated by 8% SDS-PAGE gels. *A*, immunoblotting was carried out on total proteins (20 μg) collected from the cell lysates before the immunoprecipitation assay (total proteins). The signal for the housekeeping protein GAPDH is shown below each blot. *B*, immunoblotting of “IP-proteins” was carried out after eluting the protein complexes from the beads. All immunoblots were carried out in parallel under the same transfection and extraction conditions. Western blotting was carried out with either anti-Ca_V_β3 (Alomone; catalog no.: ACC008, 1:10,000 dilution), anti-Ca_V_β2a (Alomone; catalog no.: ACC105, 1:1000 dilution), anti-Ca_V_1.2 directed against Ca_V_α1C (Alomone; catalog no.: ACC003, 1:250 dilution), anti-Ca_V_α2δ1 (Alomone; catalog no.: ACC015, 1:1000 dilution), and GAPDH (Sigma; 1:10,000 dilution) with an anti-rabbit as secondary antibody (Jackson ImmunoResearch; 1:10,000 dilution). Signals were detected with the enhanced chemiluminescence substrate. Blots were visualized with the ChemiDoc Touch system (Bio-Rad). Molecular weights were estimated using Image Lab software, version 5.2 (Bio-Rad) by linear regression of standard molecular weight markers. GAPDH, Ca_V_β3, Ca_V_β2a, Ca_V_α2δ1, and Ca_V_1.2 proteins migrated (in kilodalton) at 35, 60, 80, 175, and 250 kDa, respectively. From *left* to *right* in *A* and *B*: lane 1: Ca_V_1.2 WT + Ca_V_α2δ1 + Ca_V_β2a; lane 2: Ca_V_1.2 G449R + Ca_V_α2δ1 + Ca_V_β2a; lane 3: Ca_V_1.2 WT + Ca_V_α2δ1 + Ca_V_β3; and lane 4: Ca_V_1.2 G449R + Ca_V_a2δ1 + Ca_V_β3. HEKT, human embryonic kidney 293T cell line; IP, immunoprecipitation.

It is well known that gain-of-function mutations G402S and G406R (Fig. 1) decelerate inactivation kinetics (20, 21, 32, 33, 34). In contrast, the inactivation kinetics of the gain-of-function TS Ca_V_1.2 G419R variant classified as a pathogenic TS variant (35) were slightly faster than Ca_V_1.2 WT (28). The faster inactivation kinetics were associated with increased peak current density and a leftward shift in the voltage of activation, leading to an increased probability of channel being open at physiological voltages without any significant change in the voltage dependence of inactivation (Table 1). Glycine residues close to the pore (*e.g.*, Gly402 and Gly406) appear to be essential to convey the movement of the inactivation gate, whereas inserting glycine residues further away and closer to the high-affinity binding domain for Ca_V_β (Fig. 1) yielded opposite results (29, 30). Increased flexibility within this stretch has been argued to loosen up the interaction between Ca_V_β and Ca_V_1.2 (30). We validated that the substitution of the glycine residue at position 449 (rabbit numbering) does not impair the interaction with the canonical Ca_V_β and Ca_V_α2δ1 subunits (Fig. 2). The latter observation is in line with the recent demonstration that interaction with Ca_V_α2δ1 involves extracellular loops of Ca_V_1.2 (4, 36, 37). We thus turned to investigate functional regulation by the ubiquitous CaM (38). Disease-causing mutations at CaM proteins lead to major cardiac dysfunction, and in turn, mutations at the CaM-binding site of ion channels have been associated with a host of diseases (39).

**Table 1:** Electrophysiological properties of Ca_V_1.2 WT and G449R with W-13

Ca_v_1.2	CaM	n/N	Electrophysiological properties				
			Peak I (pA/pF)	E_0.5,act_ (mV)	R100	n/N	E_0.5,inact_ (mV)
WT	Native	30/7	−15 ± 4	−10 ± 3	0.65 ± 0.04	14/5	−33 ± 3
	+W-13	17/4	−8 ± 2 *p* = 0.002 *versus* control	−12 ± 4	0.57 ± 0.02 *p* < 0.001 *versus* control	6/3	−33 ± 3
G449R	Native	31/4	−33 ± 12 *p* < 0.001 *versus* WT	−17 ± 3 *p* < 0.001 *versus* WT	0.52 ± 0.03 *p* < 0.001 *versus* WT	13/4	−35 ± 3
	+W-13	12/1	−38 ± 8	−17 ± 3	0.51 ± 0.03	5/2	−34 ± 2

Effects of CaM inhibitor W-13 on the gating properties of Ca_V_1.2 WT or G449R channels with native or endogenous CaM. Ca_V_1.2 WT or G449R were coexpressed in HEKT cells with Ca_V_β2a and Ca_V_α2δ1. Whole-cell currents were measured in the presence of 2 mM Ca^2+^ in the extracellular medium. E_0.5,inact_ values were estimated after a 5 s long depolarizing pulse to 0 mV. Fractional currents were fitted to Boltzmann equations as described in the Experimental procedures section. The R100 values report the relative current decay observed 100 ms after the peak current. n/N refers to the number of cells/transfections measured in each condition of study. Mean ± SD are shown. Statistical analysis was carried out by one-way ANOVA and a Bonferroni post hoc test.

### CaM antagonist W-13 blocks Ca_V_1.2 WT but not G449R whole-cell currents

**Figure 3:**
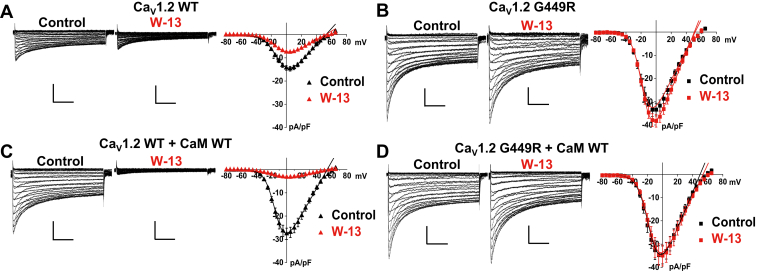
**Ca**_**V**_**1.2 G449R is insensitive to W-13.***A*, representative Ca_V_1.2 WT current traces recorded in the presence of native/endogenous CaM from HEKT cells before (*left*) and after (*middle*) the application of W-13. Peak current densities of Ca_V_1.2 WT currents are plotted against the applied voltages and fitted by a Boltzmann equation (*right*). Incubation with 10 μM W-13 for 15 min reduced the Ca_V_1.2 WT current density by approximately 50%, from −15 ± 4 pA/pF under control conditions *versus* −8 ± 2 pA/pF in the presence of W-13. *B*, representative Ca_V_1.2 G449R current traces recorded in the presence of native/endogenous CaM from HEKT cells before (*left*) and after (*middle*) W-13 treatment. In contrast to Ca_V_1.2 WT, Ca_V_1.2 G449R currents were unaffected by the W-13 and did not display any inhibition in the peak current density (*right*). *C*, representative Ca_V_1.2 WT current traces cotransfected with CaM WT recorded from HEKT cells before (*left*) and after (*middle*) W-13 treatment. Overexpression of CaM WT significantly enhanced the current density of Ca_V_1.2 WT, whereas only approximately, 10% of peak Ca_V_1.2 currents remained following W-13 treatment (*right*). Peak current densities of Ca_V_1.2 WT coexpressed with CaM WT are plotted against the applied voltages and fitted by a Boltzmann-like equation. *D*, representative Ca_V_1.2 G449R current traces cotransfected with CaM WT recorded from HEKT cells before (*left*) and after (*middle*) application of W-13. Unlike the Ca_V_1.2 WT channels, overexpression of CaM WT did not alter the Ca_V_1.2 G449R currents. Furthermore, inhibition of the Ca_V_1.2 G449R peak currents by W-13 was undetectable (*right*). The *vertical scale bars* are 10 pA/pF, and the *horizontal scale bars* are 100 ms throughout. All biophysical values are reported in Table 1, Table 2, Table 3. CaM, calmodulin; HEKT, human embryonic kidney 293T cell line.

Functional regulation of Ca_V_1.2 WT by endogenous CaM was examined with the membrane-permeable naphthalenesulfonamide derivative CaM antagonist W-13. Under our conditions, Ca_V_1.2 WT currents activated at −35 mV and reached the peak inward current at +5 mV. As seen in Figure 3*A*, the peak current density of Ca_V_1.2 WT was reduced by about 50% from −15 ± 4 pA/pF to −8 ± 2 pA/pF after adding 10 μM W-13 into the bath. Decay of the Ca_V_1.2 WT current was accelerated in the presence of W-13, which reduced the noninactivating component of Ca_V_1.2 at the end of 100 ms depolarization (R100) from 0.65 ± 0.04 to 0.57 ± 0.02 (*p* < 0.001) (Table 1). Of note, W-13 did not impair Ca^2+^-dependent facilitation in cardiac cells (40). Under the same experimental conditions, the inhibitory effect of W-13 on the current amplitude and the acceleration of current decay were blunted in the G449R construct with −33 ± 12 *versus* −38 ± 8 pA/pF (Fig. 3*B*) suggesting that the glycine substitution prevents the channel modulation by endogenous CaM.

### CaM promotes the activity of Ca_V_1.2 WT

In a typical cellular environment, CaM targets could far exceed that of free endogenous CaM (41, 42). To further explore the regulation by CaM, CaM WT was overexpressed along with the complementary DNA (cDNA) coding for the channel subunits. Overexpressing CaM has been shown to compete with endogenous CaM WT and was successfully used to reveal the mechanistic actions of CaM on voltage-activated Ca^2+^ channels (43, 44, 45, 46). Representative current traces from cells coexpressing Ca_V_1.2 WT and CaM WT are shown in Figure 3*C*. As seen, under these conditions, the peak current density nearly doubled up from −15 ± 4 to −28 ± 8 pA/pF (*p* < 0.001 as compared with endogenous CaM) to reach values not significantly different than G449R under the same conditions (*p* > 0.05). CaM WT shifted the E_0.5,act_ to hyperpolarized potentials (*p* < 0.05) and slightly accelerated the inactivation kinetics (Table 2). CaM enhanced the fraction of Ca_V_1.2 WT currents that was inhibited by W-13, with about 90% reduction in peak current density, from 28 ± 8 pA/pF for control *versus* 3.3 ± 0.7 pA/pF for W-13 (*p* = 0.001). Overexpressing CaM WT caused undetectable changes in the peak current density, the voltage of activation, and the current decay of G449R that remained unaffected by the W-13 treatment (Fig. 3*D*) (Table 3). G449R functionally behaved like it intrinsically adopted a maximally active mode (47).

**Table 2:** Electrophysiological properties of Ca_V_1.2 WT with CaM WT and phosphorylation surrogates

Ca_V_1.2	CaM	n/N	Electrophysiological properties		
			Peak current density (pA/pF)	E_0.5,act_ (mV)	R100
Ca_V_1.2 WT	CaM WT	28/6	−28 ± 8 *p* < 0.001 *versus* native CaM	−14 ± 3 *p* = 0.002 *versus* native CaM	0.60 ± 0.03 *p* < 0.001 *versus* native CaM
	+W-13	9/2	−3.3 ± 0.7 *p* = 0.001 *versus* control	−14 ± 3	0.65 ± 0.03 *p* = 0.002 *versus* control
	CaM T79A	18/2	−13 ± 5 *p* < 0.001 *versus* CaM WT, T79D, S81D	−13 ± 2	0.69 ± 0.03 *p* < 0.01 *versus* native CaM *p* < 0.01 *versus* CaM T79D, S81D
	+W-13	16/2	−14 ± 3	−14 ± 3	0.56 ± 0.03 *p* < 0.001 *versus* control
	CaM T79D	15/4	−29 ± 7 *p* < 0.001 *versus* native CaM *p* < 0.001 *versus* CaM T79A, S81A	−15 ± 3 *p* < 0.001 *versus* native CaM	0.55 ± 0.03 *p* < 0.001 *versus* native CaM *p* < 0.001 *versus* CaM WT, T79A, S81A, S81D
	+W-13	10/2	−4 ± 1 *p* < 0.001 *versus* control	−12 ± 2	0.71 ± 0.02 *p* < 0.001 *versus* control
	CaM S81A	17/2	−13 ± 4 *p* < 0.001 *versus* CaM WT, T79D, S81D	−13 ± 2	0.66 ± 0.03 *p* < 0.01 *versus* CaM WT, T79D, S81D
	+W-13	8/1	−12 ± 2	−9 ± 3	0.61 ± 0.02 *p* = 0.02 *versus* control
	CaM S81D	14/1	−32 ± 8 *p* = 0.001 *versus* native CaM *p* = 0.001 *versus* CaM T79A, S81A	−13 ± 3	0.62 ± 0.02 *p* < 0.01 *versus* CaM T79A, T79D, S81A
	+W-13	9/1	−5 ± 1 *p* < 0.001 *versus* control	−12 ± 2	0.70 ± 0.02 *p* < 0.001 *versus* control

Effects of CaM inhibitor W-13 on the biophysical properties of Ca_V_1.2 WT channels. Ca_V_1.2 WT was coexpressed with Ca_V_β2a, Ca_V_α2δ1, and CaM WT or phosphoresistant and phosphomimetic variants (T79A, T79D, S81A, or S81D). Activation properties (E_0.5,act_) were estimated from the *I–V* relationships and fitted as described in the Experimental procedures section. The R100 values report the relative current decay observed 100 ms after the peak current. n/N refers to the number of cells/transfections measured in each condition of study. Mean ± SD are shown. Statistical significance of observed differences was evaluated using one-way ANOVA and Bonferroni test (*p* < 0.05). As seen, coexpression with CaM WT, CaM T79D, or T81D potentiated Ca^2+^ currents that were sensitive to CaM antagonists and sped up current decay. In contrast, coexpression with CaM T79A or CaM S81A (phosphoresistant analogs) produced Ca^2+^ currents similar to the ones measured in the presence of endogenous/native CaM in terms of peak current density and current decay. Nonetheless, CaM T79A and CaM S81A did not prevent the robust activation and rendered Ca^2+^ currents insensitive to inhibition by W-13.

**Table 3:** Electrophysiological properties of Ca_V_1.2 G449R with CaM WT and phosphorylation surrogates

Ca_v_1.2	CaM	n/N	Electrophysiological properties		
			Peak current density (pA/pF)	E_0.5,act_ (mV)	R100
Ca_V_1.2 G449R	CaM WT	20/3	−35 ± 10	−16 ± 2	0.50 ± 0.03
	+W-13	9/2	−35 ± 9	−17 ± 3	0.54 ± 0.01 *p* = 0.04 *versus* control
	CaM T79A	15/2	−32 ± 10	−16 ± 2	0.50 ± 0.02
	+W-13	14/2	−33 ± 10	−17 ± 3	0.45 ± 0.03 *p* < 0.001 *versus* control
	CaM T79D	24/3	−35 ± 9	−18 ± 4	0.50 ± 0.02
	+W-13	8/1	−32 ± 9	−16 ± 2	0.50 ± 0.02
	CaM S81A	13/1	−34 ± 9	−18 ± 2	0.48 ± 0.02 *p* = 0.002 *versus* native CaM *p* = 0.02 *versus* CaM S81D
	+W-13	9/1	−33 ± 7	−19 ± 2	0.51 ± 0.02
	CaM S81D	9/1	−35 ± 8	−17 ± 3	0.52 ± 0.02 *p* = 0.02 *versus* CaM S81A
	+W-13	10/1	−33 ± 10	−15 ± 3	0.50 ± 0.02

Effects of CaM inhibitor W-13 on the biophysical properties of Ca_V_1.2 G449R channels. Ca_V_1.2 G449R was coexpressed with Ca_V_β2a, Ca_V_α2δ1, and CaM WT or phosphoresistant and phosphomimetic variants (T79A, T79D, S81A, or S81D). Activation properties (E_0.5,act_) were estimated from the *I–V* relationships and fitted as described in the Experimental procedures section. The R100 values report the relative current decay observed 100 ms after the peak current. n/N refers to the number of cells/transfections measured in each condition of study. Mean ± SD are shown. Statistical analysis was evaluated using one-way ANOVA and Bonferroni post hoc test. As seen, all experimental conditions yielded whole-cell Ca^2+^ currents that were not significantly different from one another (*p* > 0.05).

**Figure 4:**
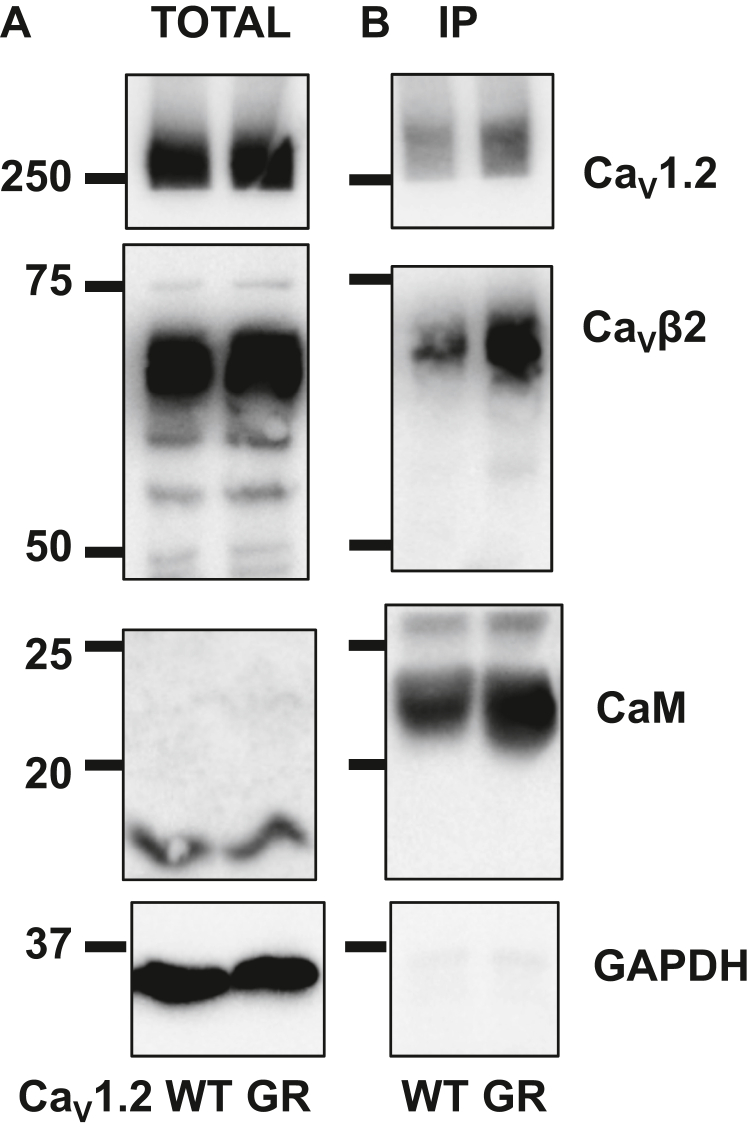
**Calmodulin (CaM) pulls down the L-type calcium channel.** HEKT cells were transiently transfected with Ca_V_β2a, CaM WT, and either Ca_V_1.2 WT (WT) or G449R (GR). CaM was captured by the anti-His–coated beads. *A*, proteins were homogenized, and a fraction of this solution (referred to as total) was set aside to validate protein expression. *B*, coimmunoprecipitation was carried out with anti-His magnetic beads. The bound proteins were eluted (referred to as pull-down) and electrophoresed on a 6% SDS-polyacrylamide gel or a 10% SDS-polyacrylamide gel for CaM and GAPDH before being transferred onto a nitrocellulose membrane. Western blotting was carried out with anti-Ca_V_β2 (Alomone; catalog no.: ACC105, 1:1000 dilution), anti-Ca_V_1.2 (Alomone; catalog no.: ACC003, 1:250 dilution) with an anti-rabbit as secondary antibody (Jackson ImmunoResearch, 1:10,000 dilution), and anti-CaM (Millipore; catalog no.: 05-193, 1:1000 dilution) with an anti-mouse as secondary antibody (Jackson ImmunoResearch, 1:10,000 dilution). Molecular weights were estimated using Image Lab software, version 5.2 (Bio-Rad) by linear regression of standard molecular weight markers. Ca_V_1.2 WT and G449R, Ca_V_β2a, and CaM proteins were translated at the expected molecular masses of 250, 70, and 18 to 24 kDa, respectively. Ca_V_1.2 WT and G449R were successfully pulled indicating that Ca_V_1.2 G449R interacts with Ca_V_β2a and CaM. This result was successfully obtained from four independent transfections carried out over the course of 3 months. HEKT, human embryonic kidney 293T cell line.

**Figure 5:**
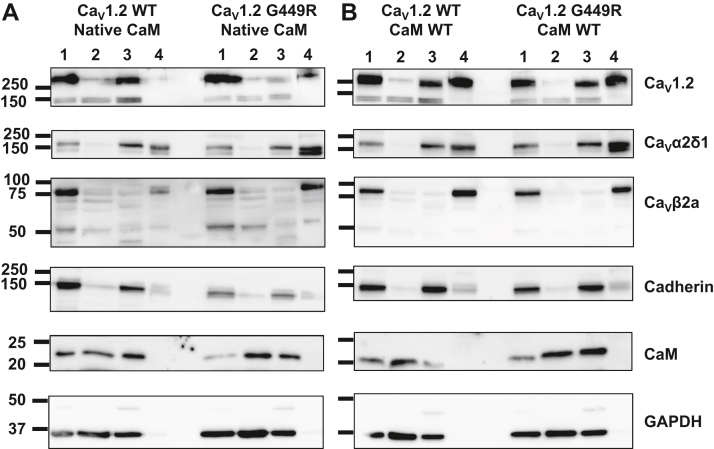
**CaM promotes the cell surface localization of Ca**_**V**_**1.2 WT but not Ca**_**V**_**1.2 G449R.***A*, HEKT cells were transiently transfected with Ca_V_1.2 WT + Ca_V_α2δ1 + Ca_V_β2a (*left*) and Ca_V_1.2 G449R + Ca_V_α2δ1 + Ca_V_β2a (*right*) in the presence of native/endogenous CaM. *B*, HEKT cells were transiently transfected with Ca_V_1.2 WT + Ca_V_α2δ1 + Ca_V_β2a + CaM WT (*left*) and Ca_V_1.2 G449R + Ca_V_α2δ1 + Ca_V_β2a + CaM WT (*right*). Two days after transfection, the cells were lysed, and cell fractions were obtained through preparative ultracentrifugation as described in the Experimental procedures section. Western blotting was carried out for the four protein fractions found in lanes 1 to 4; lane 1: total proteins; lane 2: cytoplasmic proteins; lane 3: total membrane proteins; and lane 4: plasma membrane proteins. The proteins were probed with the following antibodies: Ca_V_1.2 (Alomone; catalog no.: ACC003, 1:250 dilution) with anti-rabbit (1:10,000 dilution); Ca_V_α2δ1 (Alomone; catalog no.: ACC015, 1:1000 dilution) with anti-rabbit (1:10,000 dilution); Ca_V_β2a (Alomone; catalog no.: ACC105, 1:1000 dilution) with anti-rabbit (1:10,000 dilution); CaM (Millipore; catalog no.: 05-193, 1:1000 dilution) with anti-mouse (1:10,000 dilution); His (Invitrogen; catalog no.: 71700, 1:1000 dilution) with anti-mouse (1:10,000 dilution); and cadherin (Pan-cadherin; Thermo Fisher; catalog no.: 71-7100, 1:1000 dilution) with anti-rabbit (1:10,000). Cadherin was used as a marker for the plasma membrane. The membrane was cut at 115 and 28 kDa to probe first Ca_V_1.2, Ca_V_β2a, and CaM. Membranes were then stripped and reprobed with antibodies against the proteins: Ca_V_α2δ1, cadherin, and housekeeping GAPDH (Sigma; catalog no.: G9545, 1:10,000 dilution with anti-rabbit [1:10,000 dilution]). Each lane was loaded with 20 μg proteins. The *lines* to the *left* of the blots indicate the position of the molecular markers, and the value is provided in kilodalton. The molecular masses were estimated by linear regression and interpolation from the molecular mass markers using the Image Lab software, version 5.2 (Bio-Rad). As seen in *A*, in the presence of endogenous CaM, the signal for Ca_V_1.2 WT was stronger in the total membrane protein fraction (lane 3) than in the cell surface protein fraction (lane 4). Under the same conditions, the signal for Ca_V_1.2 G449R was stronger in the cell surface protein fraction. *B*, demonstrates that under conditions where CaM was overexpressed, the signal for Ca_V_1.2 WT and Ca_V_1.2 G449R is stronger in the cell surface protein fraction. Along with Ca_V_1.2, Ca_V_α2δ1 (as previously reported (4)) and Ca_V_β2a are found in the cell surface fraction but not CaM and GAPDH. This observation suggests that the interaction of CaM with the pore-forming subunit is very robust during the cell surface export and is compatible with the binding and unbinding kinetics of CaM in other cell types (42). No significant signal was found in the cytoplasmic fraction for Ca_V_α2δ1 and the membrane-anchored Ca_V_β2a. This result was successfully obtained from two independent transfections carried out over the course of 2 months. CaM, calmodulin; HEKT, human embryonic kidney 293T cell line.

CaM was previously shown to bind to the I–II linker in addition to other intracellular sites within Ca_V_1.2 (48). Pull-down assays demonstrated that CaM is tethered to the WT and the G449R channel complex (Fig. 4). In fact, the protein signal for G449R appeared to be more intense, hinting that it could maintain a stronger interaction with CaM. Enhanced channel activity could arise because of improved activation gating and/or increase in the relative cell surface protein expression/stability. Previous studies have reported that CaM enhances trafficking of Ca_V_1.2 in human embryonic kidney (HEK) cells (49). CaM-induced increases in peak current density may reflect an improved surface expression of channel complexes. To sort this issue, we performed a series of cell fractionation assays. As seen in Figure 5 in the presence of endogenous CaM, the signal for Ca_V_1.2 WT was stronger in the total membrane protein fraction (Fig. 5*A*, lane 3) than in the cell surface protein fraction (Fig. 5*A*, lane 4). Under the same conditions, the signal for Ca_V_1.2 G449R was stronger in the cell surface protein fraction suggesting that G449R is better trafficked or more stable than channel complexes including the WT protein and endogenous CaM. Differences in the relative channel expression were obliterated when the channel complexes were overexpressed with CaM WT (Fig. 5*B*). Under these conditions, the WT and G449R channel complexes are similarly found in the cell surface fraction. Overexpression of CaM enhanced the cell surface trafficking of Ca_V_1.2 WT, which can account in part for the increased peak current density and possibly the increase in the activation gating.

### Phosphomimetic surrogates of CaM are associated with increased activity of Ca_V_1.2 WT

The precise mechanism though which W-13 inhibits CaM is not currently known, but it has been shown that W-13 bends the flexible linker of CaM between Met78 and Glu82 (50, 51), a region that harbors two important phosphorylation sites Thr79 and Ser81 (52). Phosphorylation at these two sites causes structural changes in the relative orientation of the C- and N-lobes, which in turn modulate the interaction of CaM with its protein targets (53, 54). To evaluate the structural properties of the flexible linker, we introduced phosphomimetic and phosphoresistant mutations on CaM by individually changing phosphorylation sites Thr79 and Ser81 to alanine (A) or aspartate (D), respectively, the latter mimicking the negative charge change induced by post-translational modification.

**Figure 6:**
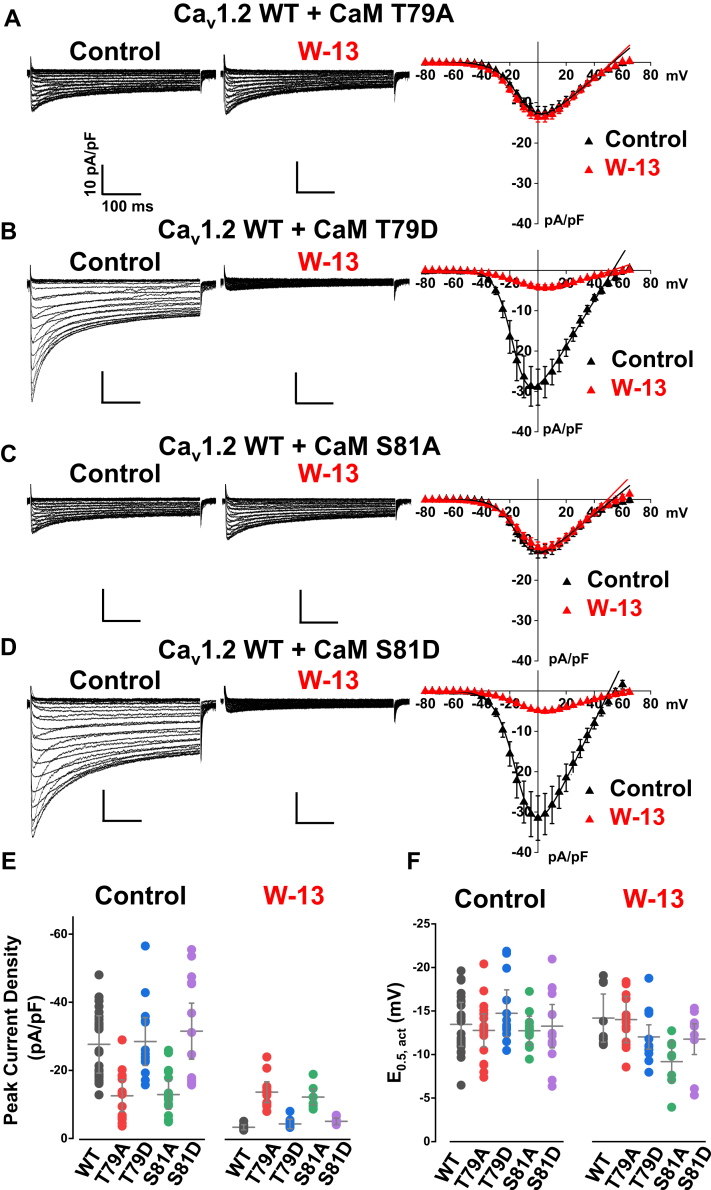
**Phosphomimetic CaM T79D and S81D upregulate Ca**_**V**_**1.2 WT channels.***A* and *C*, *middle*, Ca_V_1.2 WT was coexpressed with the “phosphoresistant” CaM mutations T79A or S81A. Overexpression of CaM T79A or S81A failed to enhance the currents and was insensitive to W-13. *A* and *C*, *right*, average *I–V* curves of Ca_V_1.2 WT coexpressed with CaM T79A or S81A. The peak current densities were not different between control and W-13 treatment. *B* and *D*, *left*, *middle*, Ca_V_1.2 WT current traces recorded from HEKT cells after coexpression with phosphomimetic CaM T79D or S81D. Overexpression of CaM T79D or CaM S81D boosted Ca_V_1.2 peak currents that were sharply abolished by the extracellular application of W-13. The *vertical scale bars* are 10 pA/pF, and the *horizontal scale bars* are 100 ms throughout. *B* and *D*, *right*, average *I–V* curves of Ca_V_1.2 WT coexpressed with CaM T79D or S81D for control and W-13 treatment. *E* and *F*, the distribution of the peak current densities and E_0.5,act_ for control and W-13 are summarized as *filled circles* for Ca_V_1.2 WT coexpressed with either CaM WT (*black*), T79A (*red*), T79D (*blue*), S81A (*green*), or S81D (*light purple*). The mean data ± SD are shown as *gray hyphens*. The values of the average peak current densities and E_0.5,act_ are listed in Table 2. CaM, calmodulin; HEKT, human embryonic kidney 293T cell line.

Overexpression of phosphoresistant CaM T79A (Fig. 6*A*, *left*) or CaM S81A (Fig. 6C, *left*) abrogated the upregulation of Ca_v_1.2 WT currents by CaM WT. The peak current densities of Ca_V_1.2 WT were −13 ± 5 pA/pF for CaM T79A (*p* < 0.001) and −13 ± 4 pA/pF for CaM S81A (*p* < 0.001) as compared with −28 ± 8 pA/pF when coexpressed with CaM WT (Table 2). The two phosphoresistant mutations failed to increase the peak current density and activation gating. Overexpression with phosphomimetic CaM variants T79D (Fig. 6*B*, *left*) or S81D (Fig. 6*D*, *left*) produced peak current densities and activation potentials comparable to those obtained with Ca_V_1.2 WT + CaM WT (Fig. 6, *E* and *F*). The structural properties of the flexible linker were also shown to regulate the activity of Ca^2+^-activated SK2 channels, although in this latter case, CaM T79D reduced channel activity (55).

CaM inhibitor W-13 substantially diminished Ca_V_1.2 + CaM T79D or Ca_V_1.2 + CaM S81D currents by around 85% (Fig. 6, *B* and *D*, *middle*) with values of −4 ± 1 pA/pF (Fig. 6*B*, *right*) and −5 ± 1 pA/pF, respectively (Fig. 6*D*, *right*). Treatment with W-13 did not however further inhibit Ca_V_1.2 channels coexpressed with phosphoresistant CaM T79A and S81A (Fig. 6*A* and *C*, *middle* and *right*; Fig. 6*E*) and had little impact on the activation gating under any of these conditions (Fig. 6*F* and Table 2).

The modulation of CaM WT on the function of Ca_V_1.2 WT was equivalent to the action of phosphomimetic surrogates CaM T79D and S81D, suggesting that the phosphorylated form of CaM is responsible for the functional upregulation of Ca_V_1.2. Roughly 10 to 45% of endogenous CaM is constitutively phosphorylated *in vivo* by casein kinase II (CK2) (52, 56, 57), and *in vitro* studies confirmed that CaM Thr79 and Ser81 are the most likely targets (58, 59). Experiments were thus performed in the presence of 4,5,6,7-tetrabromobenzotriazole (TBB; Tocris, Bio-Techne), a specific inhibitor of CK2. As shown in Table 4, TBB significantly decreased the peak current density by ≈70% and right shifted the activation gating of whole-cell currents recorded in the presence of Ca_V_1.2 WT with native CaM. Furthermore, TBB annihilated the impact of overexpressing CaM WT on the peak current density of Ca_V_1.2 WT. The impact of TBB was comparable to the disrupting effect of W-13 and much greater than the coexpression with either CaM T79A or S81A.

**Table 4:** Electrophysiological properties of Ca_V_1.2 WT and G449R with TBB

Ca_v_1.2	CaM	n/N	Electrophysiological properties		
			Peak current density (pA/pF)	E_0.5,act_ (mV)	R100
Ca_V_1.2 WT	Native CaM	30/7	−15 ± 4	−10 ± 3	0.65 ± 0.04
	+TBB	6/2	−4 ± 1 *p* = 0.001 *versus* control	−5 ± 1 *p* = 0.001 *versus* control	0.80 ± 0.01 *p* < 0.001 *versus* control
	CaM WT	28/6	−28 ± 8	−14 ± 3	0.60 ± 0.03
	+TBB	5/1	−4.9 ± 0.7 *p* < 0.001 *versus* control	−8 ± 2 *p* = 0.001 *versus* control	0.73 ± 0.01 *p* < 0.001 *versus* control
Ca_V_1.2 G449R	Native CaM	31/4	−33 ± 12	−17 ± 3	0.52 ± 0.03
	+TBB	7/2	−15 ± 6 *p* = 0.001 *versus* control	−15 ± 3	0.55 ± 0.03
	CaM WT	20/3	−35 ± 10	−16 ± 2	0.50 ± 0.03
	+TBB	7/2	−13 ± 5 *p* < 0.001 *versus* control	−13 ± 4 *p* = 0.06 *versus* control	0.57 ± 0.03 *p* < 0.01 *versus* control

Effects of TBB, the cell-permeable inhibitor of CK2 on the biophysical properties of Ca_V_1.2 WT and Ca_V_1.2 G449R channels. Ca_V_1.2 (WT or G449R) was coexpressed with Ca_V_β2a, Ca_V_α2δ1, and CaM WT as indicated. Two days after transfection, experiments were performed in the presence of 2.5 μM TBB, usually regarded as a membrane-permeable specific inhibitor of CK2. Activation properties (E_0.5,act_) were estimated from the *I–V* relationships and fitted as described in the Experimental procedures section. The R100 values report the relative current decay observed 100 ms after the peak current. n/N refers to the number of cells/transfections measured in each condition of the study. Mean ± SD are shown. Statistical analysis was evaluated using one-way ANOVA and Bonferroni post hoc test. As seen, TBB significantly decreased the channel peak current density under all conditions. It also significantly right shifted the activation gating of Ca_V_1.2 WT but not of Ca_V_1.2 G449R.

Ca^2+^ binding to CaM remains a prerequisite step for driving the channel complex into its higher functioning mode. Overexpression of the Ca^2+^-free form of CaM (CaM1234 or CaM D20A/D56A/D93A/D129A) decelerated, as expected, the CDI kinetics (Table 5). It also abrogated the increased peak current density and restored its activation gating to the level observed in the presence of endogenous CaM.

**Table 5:** Effect of CaM1234 on electrophysiological properties of Ca_V_1.2 WT and Ca_V_1.2 G449R

Ca_V_1.2	CaM	n/N	Electrophysiological properties		
			Peak current density (pA/pF)	E_0.5,act_ (mV)	R100
Ca_V_1.2 WT	CaM1234	10/2	−9 ± 2 *p* < 0.001 *versus* CaM WT	−6 ± 2 *p* < 0.001 *versus* CaM WT	0.76 ± 0.02 *p* < 0.001 *versus* CaM WT
	+W-13	7/1	−4 ± 2 *p* =0.04 *versus* control	−5 ± 2	0.71 ± 0.02 *p* < 0.001 *versus* control
Ca_V_1.2 G449R	CaM1234	4/2	−17 ± 4 *p* < 0.001 *versus* CaM WT	−10 ± 2 *p* < 0.001 *versus* CaM WT	0.73 ± 0.01 *p* < 0.001 *versus* CaM WT
	+W-13	6/1	−19 ± 2	−10 ± 2	0.63 ± 0.01 *p* < 0.001 *versus* control

Whole-cell currents were recorded from HEKT cells transiently transfected with Ca_V_1.2 WT or variants coexpressed with Ca_V_β2a, Ca_V_α2δ1, and CaM1234. Activation properties (E_0.5,act_) were estimated from the *I–V* relationships and fitted to a BoltzIV equation as described in the Experimental procedures section. The R100 values report the relative current decay observed 100 ms after the peak current. n/N refers to the number of cells/transfections measured in each condition of study. Mean ± SD are shown. Statistical analysis was carried out against the values measured for CaM WT. Herein “control” refers to the data collected in the presence of CaM1234 in the absence of W-13.

### The gain of function in Ca_V_1.2 G449R requires the Ca^2+^-bound CaM form

**Figure 7:**
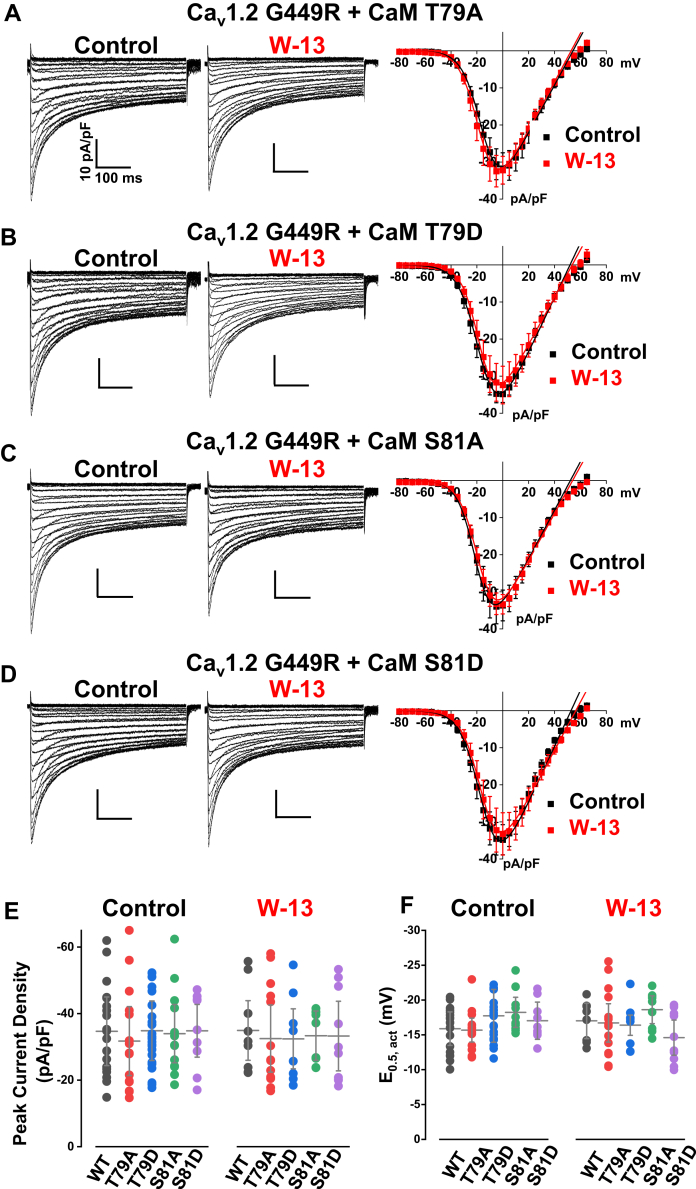
**CaV1.2 G449R is not modulated by CaM or CaM inhibitor W-13.** Representative Ca_V_1.2 G449R current traces were recorded from HEKT cells in the presence of 2 mM Ca^2+^. *A*–*D*, *left*, Ca_V_1.2 G449R was coexpressed with CaM WT, with the phosphoresistant CaM (T79A or S81A) or with phosphomimetic CaM (T79D or S81D) as shown. *A*–*D*, *middle*, Ca_V_1.2 G449R channels coexpressed with either CaM WT, T79A, T79D, S81A, or S81D are resistant to block by W-13. The *vertical scale bars* are 10 pA/pF, and the *horizontal scale bars* are 100 ms throughout. *A*–*D*, *right*, average *I–V* curves of Ca_V_1.2 G449R coexpressed with CaM T79A, T79D, S81A, or S81D. The peak current densities were not different between control and W-13 treatment. *E* and *F*, the distribution of the peak current densities and E_0.5,act_ for control conditions and after W-13 treatment are summarized individually as *filled circles* for Ca_v_1.2 G449R coexpressed with either CaM WT (*black*), T79A (*red*), T79D (*blue*), S81A (*green*), or S81D (*light purple*). The mean data ± SD are shown as *gray hyphens*. The complete set of values is found in Table 3. CaM, calmodulin; HEKT, human embryonic kidney 293T cell line.

Unlike Ca_V_1.2 WT, coexpressing either phosphoresistant CaM T79A (Fig. 7*A*, *left*) and S81A (Fig. 7*C*, *left*) or phosphomimetic CaM T79D (Fig. 7*B*, *left*) and S81D (Fig. 7*D*, *left*) with Cav1.2 G449R did not appreciably affect the peak current density, activation gating kinetics (E_0.5,act_), and current decay (R100) of Ca_V_1.2 G449R (Fig. 7, *E* and *F* and Table 3). As observed in the presence of CaM WT, the peak current densities (Fig. 7, *A*–*D*, *middle*, *right*; Fig. 7*E*), the E_0.5,act_ (Fig. 7*F*) were not altered by the application of W-13. This sharply contrasts with the results obtained with the Ca_V_1.2 WT channel complex. Nonetheless, preventing the phosphorylation of all CaM molecules with TBB reduced by 50% the peak current density measured under all other conditions (Table 4) save for CaM1234 (Table 5). Indeed, limiting Ca^2+^ binding to CaM with the CaM1234 variant not only impaired the CDI of Ca_V_1.2 G449R but also prevented the leftward shift in activation gating and the increase in peak current density (Table 5).

### Alanine substitutions in the hinge region of CaM are not disrupting interaction with Ca_V_1.2

**Figure 8:**
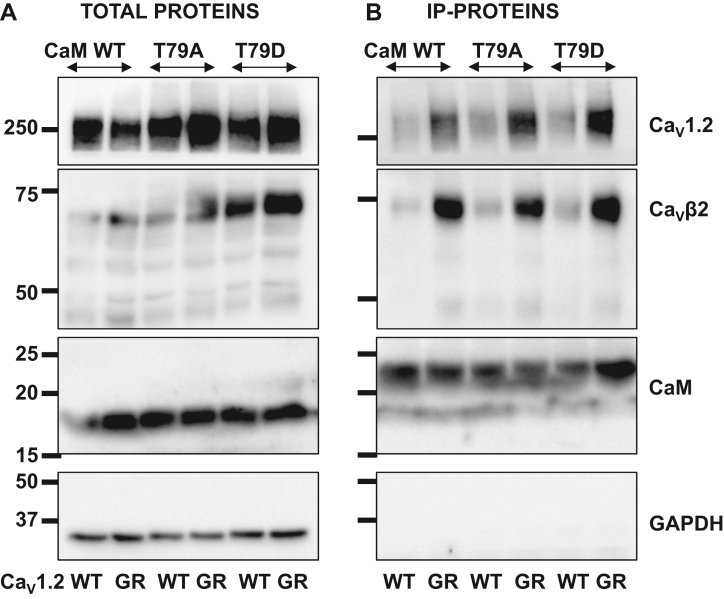
**CaM T79A and T79D coimmunoprecipitate Ca**_**V**_**1.2 WT and G449R.** HEKT cells were transiently transfected with Ca_V_β2a in the presence of Ca_V_1.2 WT or Ca_V_1.2 G449R and either CaM WT, CaM T79A, or CaM T79D. *A*, total proteins are shown. *B*, coimmunoprecipitation was carried out with anti-His magnetic beads. Immunoblotting was carried out after elution of the bound proteins using the antibodies described in the legend of Figure 4. As seen, Ca_V_1.2 WT and G449R, Ca_V_β2a, and CaM proteins were translated at the expected molecular masses of 250, 70, and 18 to 24 kDa, respectively. There was no significant difference between the signals measured in the presence of either CaM WT, CaM T79A, or CaM T79D. The signals were nonetheless systematically stronger for Ca_V_1.2 G449R than for Ca_V_1.2 WT despite equivalent loading and similar signals for the total proteins. Similar data were obtained from three independent transfections carried out over the course of 2 months with protein extraction carried out with digitonin or CHAPS. CaM, calmodulin; HEKT, human embryonic kidney 293T cell line.

We next evaluated whether CaM variants T79A and S81A alter the interaction of CaM with the pore-forming Ca_V_α1C subunit (Fig. 8). Whether for Ca_V_1.2 WT or Ca_V_1.2 G449R, the pull-down assays failed to reveal a correlation between the signal intensity and any of the tested CaM-substituted proteins indicating that phosphomimetic analogs of Ca^2+^-bound CaM impact channel function rather than protein interaction. Nonetheless, coimmunoprecipitation assays performed over a 1-year period consistently revealed a stronger signal for G449R proteins than for Ca_V_1.2 WT proteins suggesting that the glycine to arginine substitution at position 449 could increase the affinity of CaM for Ca_V_1.2.

### Substitution with an alpha-helix breaker in Ca_V_1.2 antagonizes channel function

**Figure 9:**
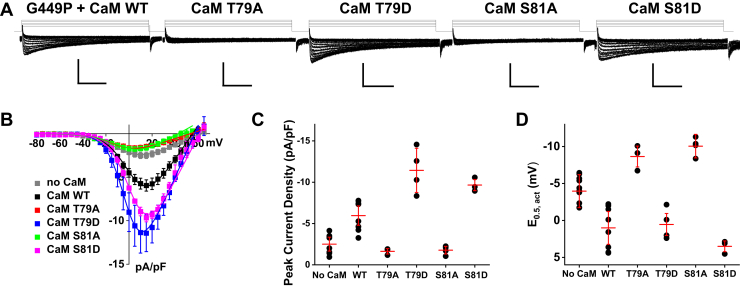
**Ca**_**V**_**1.2 G449P is modulated by CaM phosphorylation surrogates.***A*, whole-cell currents were recorded from HEKT cells transiently transfected with Ca_V_1.2 G449P coexpressed with Ca_v_β2a and Ca_v_α2δ1 and either CaM WT, with the phosphoresistant CaM (T79A or S81A), or with phosphomimetic CaM (T79D or S81D) as indicated. Exemplar traces are shown (from *left* to *right*) for Ca_V_1.2 G449P + CaM WT, G449P + CaM T79A, G449P + CaM T79D, G449P + CaM S81A, and G449P + CaM S81D. The *vertical scale bars* are 10 pA/pF, and the *horizontal scale bars* are 100 ms throughout. *B*, the corresponding peak current densities are plotted as a function of applied voltage. *C* and *D*, the summarized distribution of the peak current densities and the midpotential of activation E_0.5,act_. Peak whole-cell currents and E_0.5,act_ are reported individually as *black circles*. The mean data ± SD are shown as *red hyphens*. Values of peak current densities and E_0.5,act_ are reported in Table 6. CaM, calmodulin; HEKT, human embryonic kidney 293T cell line.

The structural properties of the I–II linker near the high-affinity binding site for Ca_V_β have been consistently shown to modulate the gating properties of Ca_V_1 and Ca_V_2 channels (31). In Ca_V_1.2, most, if not all substitutions, tested at position 449 altered the channel properties. Stronger activation gating and faster inactivation kinetics characterized Ca_V_1.2 G449A, G449D, and G449K in the presence of endogenous CaM (Fig. S1 and Table 6). All these substituted channels activated at more hyperpolarized voltages than Ca_V_1.2 WT with a threshold at −40 mV and currents peaking between 0 and −5 mV. These data could suggest that α-helix-enhancing residues and/or positively charged residues increase the channel affinity for CaM. Hence, the CaM–channel complex would be very stable in the presence of endogenous CaM as to avert the impact of CaM mutants. To test the role of the secondary structure, position 449 in Ca_V_1.2 was substituted with proline, recognized as α-helix breaker (60). G449P produced whole-cell peak currents (−2.5 ± 0.8 pA/pF, n = 10, N = 2, *p* < 0.001 *versus* Ca_V_1.2 WT) that were five times smaller than Ca_V_1.2 WT but significantly different than voltage-activated inward Ca^2+^ currents measured in nontransfected cells. The activation gating of G449P was right shifted when compared with Ca_V_1.2 WT. In contrast to G449R and G449K, Ca_V_1.2 G449P was modulated by CaM phosphomimetic variants (Fig. 9 and Table 6). Peak currents of G449P nearly tripled in the presence of CaM WT, CaM T79D, or CaM S81D and were not significantly altered by coexpressing CaM T79A or CaM S81A (Fig. 9, *A* and *B*). Remarkably, the activation of the G449P channel was left shifted in the presence of the phosphor-silenced CaM variants (Fig. 9*D*), the only occurrence where the larger peak currents were not associated with stronger activation gating. Altogether, these observations support a strong mechanistic link between the structural properties of the I–II linker near the binding site for Ca_V_β and the modulation of the channel activation gating by CaM. In particular, the channel propensity to adopt a longer α-helix in this region appears to improve the activation gating of the channel and to supersede the modulation by the phosphorylated forms of CaM.

**Table 6:** Electrophysiological properties of Ca_V_1.2 Gly449 variants with CaM phosphorylation surrogates

Ca_V_1.2	CaM	n/N	Electrophysiological properties		
			Peak current density (pA/pF)	E_0.5,act_ (mV)	R100
Ca_V_1.2 G449A	Native	12/2	−35 ± 8	−18 ± 3	0.55 ± 0.03
	CaM WT	22/4	−31 ± 8	−15 ± 2	0.57 ± 0.03
	CaM T79A	7/1	−11 ± 2 *p* < 0.001 *versus* native CaM *p* < 0.001 *versus* CaM WT	−13 ± 2 *p* = 0.002 *versus* native CaM	0.70 ± 0.01 *p* < 0.001 *versus* native CaM *p* < 0.001 *versus* CaM WT
	CaM T79D	6/1	−28 ± 8	−18 ± 2	0.55 ± 0.02
	CaM S81A	13/2	−14 ± 3 *p* < 0.001 *versus* native CaM *p* < 0.001 *versus* CaM WT	−13 ± 2 *p* < 0.001 *versus* native CaM	0.70 ± 0.03 *p* < 0.001 *versus* native CaM *p* < 0.001 *versus* CaM WT
	CaM S81D	19/2	−30 ± 9	−14 ± 3 *p* = 0.007 *versus* native CaM	0.58 ± 0.03
Ca_V_1.2 G449D	Native	17/2	−14 ± 4	−15 ± 2	0.62 ± 0.03
	CaM WT	17/2	−27 ± 8 *p* < 0.001 *versus* native CaM	−17 ± 2	0.56 ± 0.02 *p* < 0.001 *versus* native CaM
	CaM T79A	16/1	−15 ± 4 *p* < 0.001 *versus* CaM WT	−16 ± 3	0.59 ± 0.02 *p* = 0.04 *versus* native CaM *p* = 0.002 *versus* CaM WT
	CaM T79D	14/1	−29 ± 6 *p* < 0.001 *versus* native CaM	−18 ± 2 *p* = 0.007 *versus* native CaM	0.55 ± 0.02 *p* < 0.001 *versus* native CaM
	CaM S81A	21/2	−16 ± 4 *p* < 0.001 *versus* CaM WT	−15 ± 2	0.60 ± 0.02 *p* < 0.001 *versus* CaM WT
	CaM S81D	10/1	−33 ± 7 *p* < 0.001 *versus* native CaM	−18 ± 2 *p* = 0.03 *versus* native CaM	0.52 ± 0.02 *p* < 0.001 *versus* native CaM *p* = 0.01 *versus* CaM WT
Ca_V_1.2 G449P	Native	10/2	−2.5 ± 0.8	−4 ± 1	0.72 ± 0.02
	CaM WT	8/2	−6 ± 1 *p* < 0.001 *versus* native CaM	1.0 ± 2 *p* < 0.001 *versus* native CaM	0.65 ± 0.02 *p* < 0.001 *versus* native CaM
	CaM T79A	3/1	−1.6 ± 0.3 *p* = 0.001 *versus* CaM WT	−9 ±1 *p* = 0.04 *versus* native CaM *p* = 0.002 *versus* CaM WT	0.76 ± 0.01 *p* < 0.001 *versus* CaM WT
	CaM T79D	4/1	−11 ± 3 *p* < 0.001 *versus* native CaM, CaM WT	−0.5 ± 1.5 *p* =0.01 *versus* native CaM	0.57 ± 0.01 *p* < 0.001 *versus* native CaM *p* < 0.001 *versus* CaM WT
	CaM S81A	4/1	−1.8 ± 0.5 *p* < 0.001 *versus* CaM WT	−10 ± 2 *p* < 0.001 *versus* native CaM *p* < 0.001 *versus* CaM WT	0.73 ± 0.02 *p* < 0.001 *versus* CaM WT
	CaM S81D	3/1	−9.7 ± 0.7 *p* < 0.001 *versus* native CaM *p* = 0.01 *versus* CaM WT	3.5 ± 0.7 *p* < 0.001 *versus* native CaM *p* = 0.01 *versus* CaM WT	0.69 ± 0.03
Ca_V_1.2 G449K	Native	18/2	−23 ± 5	−15 ± 3	0.60 ± 0.02
	CaM WT	26/6	−26 ± 6	−15 ± 3	0.61 ± 0.03
	CaM T79A	8/1	−28 ± 7	−17 ± 2	0.57 ± 0.02 *p* = 0.006 *versus* CaM WT
	CaM T79D	12/1	−30 ± 6 *p* = 0.02 *versus* native CaM *p* = 0.002 *versus* CaM WT	−17 ± 2	0.54 ± 0.02 *p* < 0.001 *versus* native CaM *p* < 0.001 *versus* CaM WT
	CaM S81A	15/2	−27 ± 7	−16 ± 3	0.56 ± 0.03 *p* < 0.001 *versus* native CaM *p* < 0.001 *versus* CaM WT
	CaM S81D	15/2	−29 ± 5 *p* = 0.01 *versus* CaM WT	−17 ± 3	0.56 ± 0.03 *p* < 0.001 *versus* native CaM *p* < 0.001 *versus* CaM WT

Ca_V_1.2 Gly449 variants were coexpressed with Ca_V_β2a, Ca_V_α2δ1, and CaM WT or CaM T79A, CaM T79D, CaM S81A, or CaM S81D. Activation properties (E_0.5,act_) were estimated from the *I–V* relationships as described in the Experimental procedures section. n/N refers to the number of cells/transfections measured in each condition of study. Mean ± SD are shown. Statistical analysis was carried out against CaM WT or against endogenous/native CaM. As seen, Ca_V_1.2 WT, G449D, and G449P were modulated by CaM phosphovariants, whereas the properties of G449K remained unaffected. G449A was not upregulated by CaM WT but was downregulated by phosphoresistant CaM variants.

## Discussion

### Ca^2+^–CaM modulates the activity of L-type Ca_V_1.2 through multifaceted mechanisms

The ubiquitous multifunctional Ca^2+^-binding protein CaM is a two-lobe protein with each of two hydrophilic pockets for Ca^2+^ sensing separated by a flexible central linker. It is regulating the function of many voltage-gated ion channels, such as Kv7.2 (61), Na_V_1.4 (7), and in particular, voltage-gated Ca_V_ channels (7, 62) (for review, see Ref. (62)). At least two CaM molecules can simultaneously bind to the C-terminal region of Ca_V_1.2 (63, 64), but additional binding sites in the N-terminal region and the first intracellular linker of Ca_V_α1C have been identified (48, 63, 65, 66, 67). The overall structural organization of CaM within the Ca_V_1.2 channel complex remains to be established. CaM-binding sites were not resolved in the cryo-electron microscopy structure of the homologous Ca_V_1.1 channel (3).

In Ca_V_1.2 channels, Ca^2+^ binding to CaM contributes to CDI and Ca^2+^-dependent facilitation (7, 8, 9). Either process requires the binding of incoming Ca^2+^ ions to CaM preassociated to the isoleucine–glutamine motif in the C-terminal region of the pore-forming Ca_V_α1C subunit (10, 11, 12, 13). The potentiating form of CaM-dependent facilitation or upregulation is observed in native cardiac L-type channels during trains of depolarization (68, 69) but usually not reported in recombinant systems with the intact Ca_V_1.2 WT channel (8, 9, 29). We herein report that phosphomimetic analogs of CaM stimulate Ca^2+^ influx and promotes the activation gating of Ca_V_1.2. CaM promotes the cell surface trafficking of Ca_V_1.2 and stimulates function through an increase in peak current density and a leftward shift in the activation gating. In our hands, the latter actions of CaM require Ca^2+^ as it was impaired in the presence of the constitutively Ca^2+^-free form CaM1234 where the four Ca^2+^-binding sites are invalidated. This observation is compatible with data from Kim *et al.* (70), who reported that the interaction between the CaM-bound C-terminal peptide and the I–II linker is disrupted in the complete absence of Ca^2+^. CaM1234 prevented the increase in peak current density, failed to promote channel activation gating, and as expected, slowed down the CDI kinetics by 30%. Nonetheless, Ca^2+^ binding is not sufficient to account for the wide-ranging impact of CaM on channel function. The structural properties of the flexible linker region of CaM contribute to the channel response to CaM. Coexpression with CaM T79A or CaM S81A averted the boost in peak current density (although it did not alter the activation gating). In contrast, coexpression with either CaM WT or phosphomimetic CaM T79D or CaM S81A yielded similar results suggesting that phosphorylation of either site participates to the modulation of Ca_V_1.2 by CaM. Indeed, preventing the phosphorylation of native and overexpressed CaM by incubating the cells with TBB, a membrane-permeable inhibitor of CK2, nearly abrogated channel function. Hence, Ca^2+^-bound CaM modulates the function of the Ca_V_1.2 channel complex in a fashion reminiscent of the ancillary subunits Ca_V_β and Ca_V_α2δ, which like CaM may also modulate other ion channels (71).

### Multiple mechanisms converge toward Ca_V_1.2 G449R

The missense variant, glycine to arginine, was identified from a patient with prolonged QT interval (≈500 ms) and features associated with the TS, but its heterologous expression revealed a novel phenotype where the gain of function resulted from increased peak current density, a negative shift in the activation potential, and no decrease in the channel current decay (28). The hyperactive mode of the variant expressed in HEK293T (thereafter referred to as HEKT) cells was mimicked by the coexpression of Ca_V_1.2 WT with CaM WT or phosphorylated surrogates CaM T79D or CaM S81D. The functional properties of the clinical glycine to arginine variant remained remarkably insensitive to pharmacological inhibition by W-13 and by overexpression with phosphoresistant CaM analogs (T79A and S81A). The impact of the phosphorylation of CaM appears to be limited to function. Ca_V_1.2 G449R was pulled down equally by CaM WT, T79A, and T79D. Preventing the phosphorylation of CaM with TBB, an inhibitor of CK2, significantly reduced the peak current density of Ca_V_1.2 G449R by ≈50% without a significant alteration in the channel activation voltage as compared with the control conditions. The rate-limiting factor appears to be Ca^2+^ binding to CaM. Coexpression of G449R with the CaM1234 variant not only impaired the CDI and the increased peak current density but also prevented the leftward shift in activation gating. Overexpression of the CaM1234 variant obliterated the gain in the function of Ca_V_1.2 G449R yielding an activity profile akin to Ca_V_1.2 WT in the presence of endogenous/native CaM. The stronger activity of Ca_V_1.2 G449 thus minimally requires the direct or indirect action of the Ca^2+^-bound CaM form.

These observations suggest that the higher channel activity of G449R could result from a stronger affinity for native CaM. Though not measured in this article, the affinity between the two full-length proteins can be roughly approximated by the relative intensity of the signal measured in coimmunoprecipitation assays. Within all the limitations of this exercise, the protein signal obtained for G449R in coimmunoprecipitation assays was indeed systematically stronger than the signal measured for the WT channel complex when measured under the same experimental conditions and this over the course of 12 months. This interpretation is compatible with the cell surface fractionation assays showing that G449R was more likely to be found in the cell surface fraction than the WT channel complex in the presence of endogenous CaM, whereas this differential localization was not discernable when the cells were saturated with overexpressed CaM. CaM bound to the C-terminal region of Ca_V_1.2 has been previously reported to interact in a Ca^2+^-dependent manner with the cytosolic I–II loop, where is located the glycine to arginine variant (70). It is thus conceivable that the higher “intrinsic” activity of G449R results from a stronger interaction with endogenous CaM. In this model, the cellular availability of CaM could modulate the operating window of Ca_V_1.2.

Ca_V_1.2 G449R is located in a structural region involved in activation gating (72), inactivation kinetics (73), protein stability, ubiquitination (74), and cell surface trafficking (75). The proximal segment of the first intracellular linker hosts the high-affinity binding site for Ca_V_β (76) and plays a role in networking with direct partners such as galectin (74) or Ras/Rad proteins through Ca_V_β (30, 77, 78). Glycine residues are unique in their lack of side-chain steric interference, permitting a higher flexibility to protein structures. Increasing flexibility by inserting glycine residues (29, 30) decreases channel function. In contrast, decreasing flexibility of this region by removing glycine residues promoted channel function (28, 31, 79, 80). The presence of a glycine residue proximal to the α-interacting domain in Ca_V_1.2 WT could thus explain the requirement of a stronger depolarization in Ca_V_1.2 WT *versus* G449R channels. The same position is already occupied by an arginine residue in Ca_V_2.2 (31) and Ca_V_2.3 channels whose activation is left shifted when compared with Ca_V_1.2 under the same expression conditions (81).

The high-affinity binding site of Ca_V_β adopts an α-helical structure *in vitro* (82). The relative rigidity α-helix could promote a strong van der Waals interaction between the guanylate domain of Ca_V_β and hydrophobic residues of Ca_V_1.2 (29, 83, 84). In the native protein, this α-helix breaks at the glycine located at position 449 (79). Crystallographic and circular dichroism spectroscopic studies demonstrated that the arginine substitution prolongs the α-helix (31). We also report that substitution with other α-helix-promoting residues, such as alanine (85), produced channels with strong activation properties, and from the contrary, substitution with proline, regarded as a α-helix breaker, was found to curb channel activation. The substituted channels however manifested distinct electrophysiological signatures in the presence of the phosphomimetic and phosphoresistant CaM proteins, from a complete indifference (G449K) to impaired peak current density in the presence of phosphoresistant CaM variants (G449A, G449D, G449P, and G449Q). Our data are compatible with the proposition that the longer α-helix enhances the coupling of the I–II linker with the inner pore responsible for channel activation. The intracellular linker would contribute to electromechanical coupling in Ca_V_1.2 either through its intrinsic structural properties or following interaction with CaM.

The structural properties of the clinical variant could be envisioned to facilitate the interplay between accessory CaM proteins bound onto the C terminus of Ca_V_1.2 and channel function as it was postulated for AKAP150 (86). In this context, the LQTS phenotype associated with the glycine to arginine substitution in the I–II linker could result from either process: an intrinsically stronger activation of Ca_V_1.2 that renders the channel insensitive to cellular variations in phosphorylated CaM or else a higher affinity to CaM that causes the channel to be maximally activated at near endogenous concentration of CaM.

## Experimental procedures

### Recombinant DNA techniques

The Ca_V_α1C subunit of Ca_V_1.2 (GenBank accession number: X15539), Ca_V_β2a (GenBank accession number: NM_001398773), and Ca_V_α2δ1 (GenBank accession number: NM_000722) was subcloned in commercial vectors under the control of the cytomegalovirus (CMV) promoter as described elsewhere (36, 37, 75, 87). The cDNA sequence of the rabbit clone is near identical to the human clone save for an additional 30 amino acids in its N terminus, accounting for the +30 residue shift in residue numbering. The human CaM (GenBank accession number: M27319), subcloned in pcDNA3.1 (Thermo Fisher Scientific) vector with consecutive histidine (His-His-His-His-His-His) and cMyc (Glu-Gln-Lys-Leu-Iso-Ser-Glu-Glu-Asp-Leu) tags in C-terminal region, was a gift from Dr Rémy Sauvé, Université de Montréal. The cDNA mutations of CaM were introduced in this vector. CaM is numbered as reported (88) to take into account that the mature protein lacks N-terminal Met residue. All cDNA mutations in Ca_V_α1C of Ca_V_1.2 and CaM were produced with the Q5 Site-Directed Mutagenesis Kit (New England Biolabs, Inc) according to the manufacturer’s instructions. Briefly, substitutions of nucleotides were created by incorporating the desired mutation in the center of the forward primer, and the reverse primer is designed so that the 5′ ends of the two primers anneal back to back. Following the PCR, the amplified DNA is circularized, and the template is removed with a kinase–ligase–DpnI enzyme mixture, before transformation into high-efficiency NEB DH5-α competent *Escherichia coli*. All constructs were verified by automated double-stranded sequence analysis (“Centre d’expertise et de services Génome Québec”). The protein expression at the expected molecular weight was confirmed by standard Western blot analysis for each construct.

### Gene transfection and cell culture

HEKT cells were grown using standard tissue culture conditions (5% CO_2_, 37 °C) in high-glucose Dulbecco's modified Eagle's medium supplemented with fetal bovine serum (10%), l-glutamine (2 mM), penicillin (100 U/ml), and streptomycin (10 mg/ml) as described before (36, 37, 75). Using Lipofectamine 2000 (Invitrogen), as per the manufacturer's instructions, HEKT cells (80% confluence, 35 mm petri dish) were transiently transfected with cDNA plasmids, namely pCMV-Ca_V_1.2 WT or variants (4 μg), pCMV-Ca_v_β2a (4 μg), pCMV-Ca_V_α2δ1 (4 μg), and in some experiments, pcDNA3-HisB-cMyc-CaM WT or variants (2 μg), with a weight ratio of 1:1:1:0.5 for a total of 12 to 14 μg cDNAs. The molar ratio was 7:1 for CaM and Ca_V_1.2. Unless otherwise noted, the plasmids pCMV-Ca_V_β2a, pCMV-Ca_V_α2δ1, and pcDNA3-HisB-cMyc-CaM WT are simply referred to as Ca_V_β2a, Ca_V_α2δ1, and CaM WT in the text and figures. cDNA coding for peGFP (0.2 μg) was included in the cDNA mixture as a marker of successful transfection for patch-clamp experiments (4, 81). The culture medium was changed, and cells were detached with 0.05% trypsin before being replated on 35 mm petri dishes 6 h post-transfection. Whole-cell patch clamp experiments were performed 24 to 32 h after transfection.

### Coimmunoprecipitation

HEKT cells were transiently transfected with the appropriate constructs (as indicated later), and protein extraction proceeded 2 days after transfection. Experiments described in Figure 2 were carried out as follows. HEKT cells were transiently transfected with Ca_V_1.2 WT or Ca_V_1.2 G449R with pCMV-Ca_V_α2δ1 and cMyc-tagged versions of Ca_V_β3 or Ca_V_β2a using, respectively, the pCMV-Tag5-Ca_V_β3 or the pCMV-Tag5-Ca_V_β2a plasmids. Ca_V_β acted as the bait. Cell lysates were immunoprecipitated overnight with anti-cMyc magnetic beads (Pierce Anti-c-Myc Magnetic Beads; catalog no.: 88842, Thermo Fisher Scientific) to capture the given Ca_V_β. In the experiments shown in Figures 4 and 5, the constructs were pCMV-Ca_V_β2a with pCMV-Ca_V_1.2 WT or G449R and pcDNA3-HisB-cMyc-CaM WT and used CaM as the bait. Cell lysates were immunoprecipitated overnight with anti-His magnetic beads (code no.: MBL-D29111). The procedure was otherwise similar for the three experimental groups. Two different detergents have been used to compare extraction efficiency between digitonin (a nonionic saponin detergent) and CHAPS–Na (zwitterionic detergent). Both extraction conditions have produced the same results and were thus combined, for three independent experiments over the course of 2 months. Two days after transfection, cells were homogenized in 20 mM Na–Mops (pH 7.4), 300 mM NaCl, and 1% digitonin or 0.5% CHAPS–Na, supplemented with protease inhibitors without EDTA (Thermo Fisher Scientific). Homogenates were sonicated, incubated for 1 h at 4 °C, and centrifuged at 13,000 rpm for 30 min. A fraction (20 μg) of the homogenates or starting material was set aside as representative of total proteins and was immunoblotted to confirm normal protein expression. Coimmunoprecipitation was carried out using 200 μg homogenates diluted in 150 μl of 20 mM Na–Mops (pH 7.4) and 300 mM NaCl. The 200 ± 20 μl protein solution was incubated overnight with the appropriate antibody-coated magnetic beads that were collected using a PureProteome magnetic rack (Millipore). The magnetic beads were washed three times with a buffer containing 20 mM Na–Mops (pH 7.4), 300 mM NaCl, and 0.2% digitonin or alternatively 20 mM Na–Mops (pH 7.4), 300 mM NaCl, without additional detergent for the extraction under the “CHAPS conditions.” The bound proteins were eluted with Laemmli buffer (20 μl) at 95 °C for 5 min, electrophoresed on a 6% or 10% SDS-polyacrylamide gel, and transferred onto a nitrocellulose membrane for Western blotting. Antibodies are described in the figure legends. Signals were detected with the enhanced chemiluminescence substrate. Blots were visualized with the ChemiDoc Touch system (Bio-Rad). Molecular weights were estimated using Image Lab software, version 5.2 (Bio-Rad) by linear regression of standard molecular weight markers.

### Cell surface fractionation assay

Four different protein fractions (total cell lysates, cytosolic, total membrane, and plasma membrane fractions) were prepared as explained before (4). Briefly, transfected HEKT cells cultured in 100 mm dishes were homogenized at 4 °C in a Tris-based solution containing a mixture of protease inhibitors (Sigma) at pH 7.4. The cell homogenate was aliquoted into three tubes. After a 2 h incubation period at 4 °C with 1% (v/v) Triton X-100, the first tube was centrifuged at 10,000*g* for 10 min to remove cell debris, nuclei, and mitochondria. The supernatant was kept as the total protein fraction (whole-cell lysates). The second tube was centrifuged at 200,000*g* and 4 °C for 20 min. The supernatant is referred to as the cytosolic fraction. The pellet was resuspended in homogenizing buffer containing 1% (v/v) Triton X-100. After 30 min of incubation on ice, a second centrifugation was performed at 200,000*g*. The resulting supernatant is referred to as the total membrane protein fraction. The third tube was centrifuged at 10,000*g* for 10 min. The supernatant obtained was centrifuged at 200,000*g* and 4 °C for 20 min. The pellet was resuspended in the homogenizing buffer containing 0.6 M KCl. Subsequent centrifugations were performed at 200,000*g* and 4 °C for 20 min to wash out the KCl. The final pellet was resuspended in the homogenizing buffer and is considered to be enriched in plasma membrane proteins. Proteins (20 μg) were electrophoresed on a 10% SDS-polyacrylamide gel.

### Whole-cell patch-clamp recordings and data analysis

Whole-cell Ca^2+^ currents from transfected HEKT cells were recorded using pCLAMP software 11.2 (Molecular Devices) and an Axopatch 200B amplifier (Molecular Devices). Patch electrodes were pulled from borosilicate glass (Corning; code: 8161) and heat-polished to a final resistance about 3.0 to 3.5 MΩ when filled with the intracellular solution. Whole-cell currents were low-pass filtered at 2 kHz, digitized at a sampling rate of 100 μs during acquisition, and stored on a microcomputer equipped with an AD converter (Axon Digidata 1440A; Molecular Devices). Electrodes were filled with a solution containing (in millimolar) 140 CsCl, 0.6 NaGTP, 3 MgATP, 10 EGTA, 10 Hepes, titrated to pH 7.4 with NaOH. HEKT cells were bathed in a modified Earle’s saline solution (in millimolar) as follows: 135 NaCl, 20 tetraethylammonium chloride, 2 CaCl_2_, 1 MgCl_2_, 10 Hepes, titrated to pH 7.4 with potassium hydroxide. Stock solution of the cell-permeable CaM antagonists W-13 *N*-(4-aminobutyl)-5-chloro-2-naphthalenesulfonamide and monohydrochloride (Tocris, Bio-Techne) was prepared in distilled water, diluted to its final concentration just before use, and added directly in the bath solution. Cells were incubated for 15 min prior to whole-cell recordings. A few experiments were performed in the presence of 2.5 μM TBB, a cell-permeable inhibitor of CK2. Stock solution of TBB (5 mM) was prepared in dimethylsulfoxide, diluted to its final concentration just before use, and added directly in the bath solution. Whole-cell currents were recorded 15 min after drug equilibration. All experiments were carried out at room temperature (23–25 °C). Cellular capacitance was estimated by measuring the time constant of current decay evoked by a depolarizing pulse pf 10 mV applied to the cell from a holding potential of −100 mV.

Whole-cell Ca^2+^ currents were elicited from a holding potential of −100 mV and depolarized to potentials ranging from −80 to 65 mV in 5 mV increments lasting 450 ms for each step. Ca^2+^ current densities (pA/pF) were obtained by dividing the peak current by the cell capacitance. Average *I–V* curves were obtained by plotting the peak current densities *versus* the voltage and fitted to a BoltzIV equation, which is a transformed Boltzmann function for *I–V* data of the following form:

$$I=\frac{\left(V_{m}-V_{rev}\right).G_{\max}}{1+e^{\left(V-E_{0.5.act}\right)/dx}}$$

where *I* is the current, *V*_*m*_ is the applied voltage, E_0.5,act_ is the voltage at which channels are half-maximally activated, dx is the steepness of the slope, *G*_max_ is the maximal conductance, and *V*_rev_ is the reversal potential. Steady-state activation curves were constructed by dividing the peak I–V data by the driving force. The R100 ratio of Ca_V_1.2 current was defined as the peak current remaining after a 100 ms depolarizing pulse (*I*_100ms_/*I*_peak_) and was used as an indicator of the inactivation kinetics. n/N refers to the number of cells/transfections measured in each condition of study.

The steady-state inactivation was determined using a two-step protocol in which conditioning prepulses were applied from a holding potential of −100 mV to a range of potentials from −100 to 40 mV in 10 mV increments for 5 s, immediately followed by a test pulse to 5 mV for 100 ms. For the construction of inactivation curves, the peak current amplitudes during the test pulses were normalized to the maximum peak current amplitude measured at −100 mV and plotted against the conditioning pulse. Steady-state inactivation curves were fitted to a modified Boltzmann equation:

$$I/I_{\max}=\frac{A1-A2}{1+e^{\left(V-E_{0.5,inact}\right)/dx}}+A2$$

where *I*/*I*_max_ is the relative current measured at the test pulse, A1 and A2 represent, respectively, the maximum relative current value and the fraction of the noninactivated current, *V*_*m*_ is the voltage applied during the conditioning pulse, E_0.5,inact_ is the voltage at which channels are half-maximally inactivated, and dx is the steepness of the slope.

### Data analysis and statistics

Data were analyzed using a combination of pCLAMP software 11.2, Microsoft Excel, and OriginPro 2020 (OriginLab Corporation). Data in the tables are expressed as mean ± SD. Statistical significance was determined by one-way ANOVA and Bonferroni post hoc test in OriginPro 2020. The level of statistical significance was set at *p* < 0.05.

## Data availability

All data are contained within the article.

## Supporting information

This article contains supporting information.

## Conflict of interest

The authors declare that they have no conflicts of interest with the contents of this article.

## Supporting information

Supplemental Figure S1 **Ca**_**V**_**1.2 channel activity is modulated by substitutions at position Gly449.** Panel A. Whole-cell currents were recorded from HEKT cells transiently transfected with Ca_V_1.2 WT or variants co-expressed with Ca_v_β2a and Ca_v_α2δ1. Exemplar traces are shown (from left to right) for Ca_V_1.2 WT, G449A, G449D, G449P, and G449R. Ca_V_1.2 currents were elicited from a holding potential of −100 mV and were depolarized to potentials ranging from −80 to 65 mV in 5 mV increments lasting 450 ms for each step (depicted above each series of recordings). The vertical scale bars are 10 pA/pF and the horizontal scale bars are 100 ms throughout. Panel B. Peak current densities of Ca_v_1.2 WT, G449A, G449D, G449P, and G449R currents are plotted as a function of applied voltage and fitted by a Boltzman equation described in Experimental procedures. The activation curves were generated using the same protocol as in Panel A. Panels C and D show the summarized distribution of the peak current densities and the mid-potential of activation E_0.5, act_. Peak whole-cell currents and E_0.5, act_ are reported individually as black circles. The mean data ± S.D. are shown as red hyphens. Values of peak current densities and E_0.5, act_ are reported in Table 6.
