# Supplementary figures and images for: A CACNA1C variant associated with cardiac arrhythmias provides mechanistic insights in the calmodulation of L-type Ca2+ channels
Source: J Biol Chem. 2022 Oct 21;298(12):102632. doi: 10.1016/j.jbc.2022.102632 (PMC9691931; doi:10.1016/j.jbc.2022.102632)

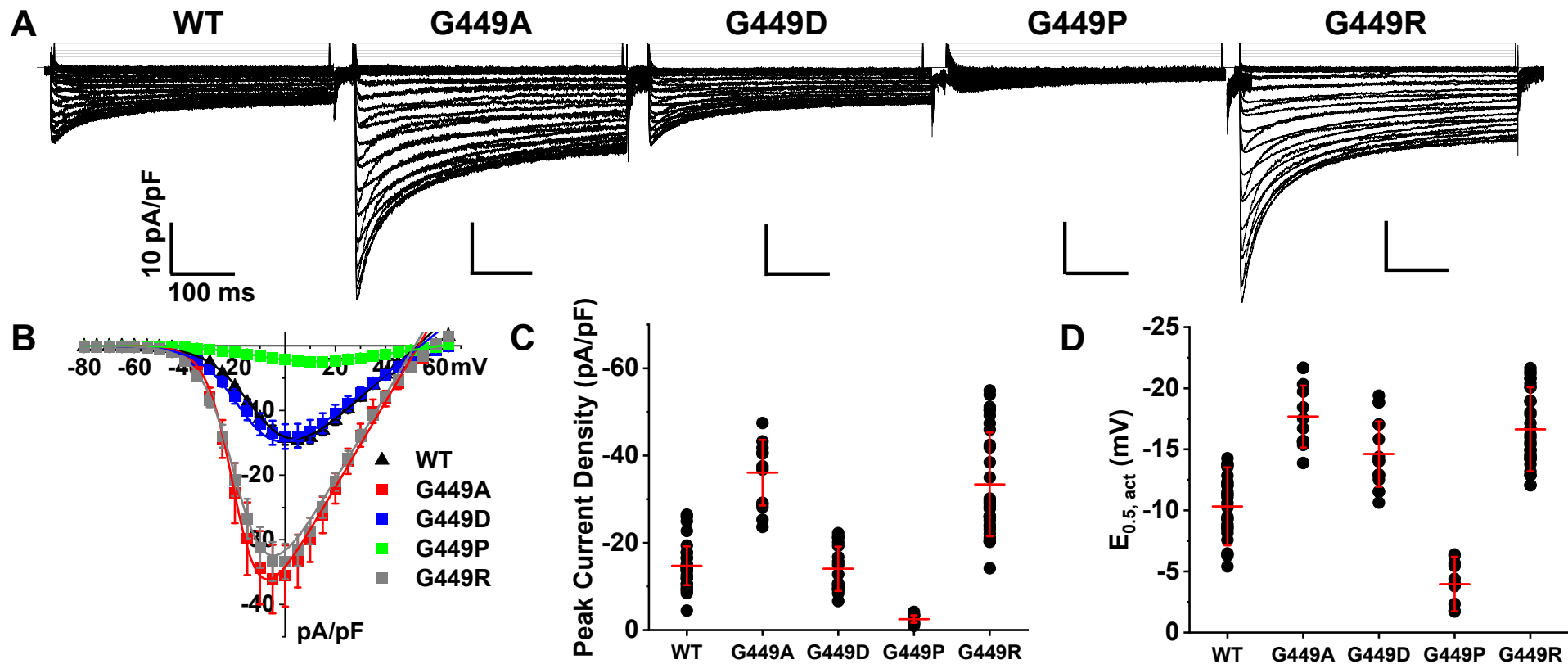

Suppl Figure

Supplement: Supplemental Figure S1 — CaV1.2 channel activity is modulated by substitutions at position Gly449. Panel A. Whole-cell currents were recorded from HEKT cells transiently transfected with CaV1.2 WT or variants co-expressed with Cavβ2a and Cavα2δ1. Exemplar traces are shown (from left to right) for CaV1.2 WT, G449A, G449D, G449P, and G449R. CaV1.2 currents were elicited from a holding potential of −100 mV and were depolarized to potentials ranging from −80 to 65 mV in 5 mV increments lasting 450 ms for each step (depicted above each series of recordings). The vertical scale bars are 10 pA/pF and the horizontal scale bars are 100 ms throughout. Panel B. Peak current densities of Cav1.2 WT, G449A, G449D, G449P, and G449R currents are plotted as a function of applied voltage and fitted by a Boltzman equation described in Experimental procedures. The activation curves were generated using the same protocol as in Panel A. Panels C and D show the summarized distribution of the peak current densities and the mid-potential of activation E0.5, act. Peak whole-cell currents and E0.5, act are reported individually as black circles. The mean data ± S.D. are shown as red hyphens. Values of peak current densities and E0.5, act are reported in Table 6. [file mmc1.pdf]
